# False Impressions

**Published:** 1888-12-01

**Authors:** Caroline Fothergill

**Affiliations:** Author of "An Enthusiast," "A Voice in the Wilderness," etc.


### False Impressions.

By Caroline Fothergill, Author of "An Enthusiast," "A Voice in the Wilderness," etc.

*(Continued from p. 127.)*

### Chapter IX.—A Step Further.

From the day of the flower show Edith felt that her life was changed; hitherto, although she had scarcely ventured to confess it even to herself, her days had passed in a monotony which amounted to dulness. She was her aunt's companion, and as that lady went out very little, Edith had perforce stayed at home too. Very few people came to the house, the number of people who sought Mrs. Lester "for her dear sake," was too insignificant to be taken into account. There were a few elderly married ladies, contemporaries of her own, who occasionally came in for a gossip, but younger women never came. If they were married they did not care for Mrs. Lester, and if they were unmarried, Mrs. Lester did not care for them; indeed, the chances were she would have sent down word that she was not at home. Edith was not encouraged, probably had she attempted it, would not have been allowed to make friends among girls of her own age, and she was too shy and fond of hiding herself in corners to induce people to draw her out and seek her acquaintance at their own houses.

She knew only one person under thirty years of age, and that was the Rev. Harry Fairfax, curate of the church which Mrs. Lester and Edith attended. Mrs. Lester had strong opinions about her responsibility as a member of the church she attended, and gave liberally to its funds, her name was therefore on the list of those who were frequently and regularly called upon by the vicar. The vicar, however, though he respected, did not like Mrs. Lester, and he more often sent Mr. Fairfax as his representative, than called himself.

Mr. Fairfax was nothing loth. He liked going. He did not like Mrs. Lester, and he never knew what to say to her, but Edith was always there and in the present state of his feelings it was happiness enough to sit and let Mrs. Lester talk and lay down parish law, while he looked at Edith and got her features, her soft wavy brown hair, and grey eyes by heart. Sometimes he talked to her, too, or rather tried to talk to her, but without much success. Naturally timid, she became doubly so in her aunt's presence, and though she had a kindly feeling for the curate, he had no especial attraction for her. She saw him when she took her class on Sunday, and on her rounds through the district, which she had undertaken at her aunt's suggestion. She knew that Mr. Fairfax looked at her a good deal, and without caring enough about it to think why he did it, she wished he would not. He in no way varied the dulness of her life, and she paid very little heed to him.

Now all this was going to be changed, life was no longer dull. There was the flower show to look back upon, and dreams to be indulged in as to whether such an event could ever happen again. Her allowance for dress too, was more than doubled, and however much she might suspect Mr. Murray's agency in the matter, she knew nothing, and so could say nothing. The very day after the flower-show George took action in the matter. He found his sister alone, and began at once.

"How much money has Edith to spend on her clothes?" he asked.

"Twenty pounds a-year," replied Mrs. Lester.

He reflected, and balanced the sum against similar sums which he spent himself. Even with the strictest economy it did not seem to him as if a good show could be made for the money, and he said promptly,

"It is not enough. Who gives it her? Does she get it from home!"

"From home!" Mrs. Lester uttered a laugh of scorn. "I doubt if they could spare her ten pounds a-year from home; you little know what her home is. I give her her allowance, and it would be amply sufficient for her requirements if she employed it properly."

"What do you mean?" asked her brother. "I cannot say that in her appearance she embodies my idea of a spendthrift."

"She is not a spendthrift in the ordinary sense of the word, but she wastes her money by sending half of it home, and stinting herself in her dress."

"What does she do that for, has she sisters? I fancied you told me not."

"She has only brothers, but her father is very poor, and she says he can give them no pocket-money, so she thinks she ought to provide for them out of her abundance.''

George frowned.

"It is all very well to wish to help them, it shows a good heart, if a deficiency in judgment; but she must not stint herself. Yesterday she was shabby, decidedly shabby, and I did not like it."

"Well, you see," said Mrs. Lester, "that was quite an exceptional occasion, and will probably not happen again. Her clothes are quite good enough for ordinary wear."

"I don't agree with you. As she is to live here she must dress in accordance with our position, and yesterday was not so exceptional an occasion as you seem to think. I found Edith a very pleasant little companion, and I shall probably take her out from time to time, it will do her good; she wants more confidence, and a girl of her age ought to see something of society. But for that she must have suitable clothes; her allowance must be increased."

"It is easy to be generous for others," said Mrs. Lester, instantly taking the alarm; "but my expenses are too heavy. I have already taxed myself in providing for Edith as I have done, and I can do no more.

George wondered within himself how she could consider giving a girl an allowance of twenty pounds a year as providing for her. He was also at a loss to see how the said twenty pounds could be a tax on an income of a thousand a-year, devoted entirely to personal expenditure. He did not, however, utter his thoughts aloud, but only said:

"Then I will increase it. Give her fifty pounds instead of twenty. You must tell her of it, and make it appear it comes from you; also give her to understand that she must not send any more home than she does now."

Mrs. Lester made some faint demur, but she saw he was right in what he said, and if the necessary change could be made without involving her in any additional expense, so much the better. George, too, would take no denial, and so she consented, and took the first opportunity of telling Edith, and probably that part of her brother's directions which she most enjoyed was that she was to make it appear as if the generosity came from her.

Edith was quite overwhelmed, and her conscience pricked her sharply for the uncharitable thoughts she had occasionally harboured against Mrs. Lester. She redoubled her attentions and lost herself in a maze of conjecture as to the meaning of her aunt's words.

"You will probably be going out more than you have hitherto done, and you will want more dresses."

Who was going to take her out? Was it possible Mr. Murray would overlook the imperfections of which she was so conscious, and take her again? The thought of the great enjoyment of that afternoon they had spent together filled her with the rosiest anticipations.

They were fulfilled. About three weeks or a month after that memorable afternoon they sat at breakfast one morning, and George opened and read his letters while Mrs. Lester and Edith exchanged the short frosty remarks which pass current for conversation early in the morning before our blood has got warmed.

Among the letters was one envelope larger than the rest, which when opened, was found to contain a card gilded and embossed, bearing the arms of the town of Mayfield. This card George studied for a few minutes, and then handed to Edith, saying:—

"What do you say to this, Edith, should you care to go?"

She read it, and then looked up with glowing cheeks.

"Oh, I should like to go very much," she said. "Will you really, do you mean—?"

She did not like to take too much for granted, and yet he must mean to go. The card was an invitation from the Mayor and Corporation to a conversazione, to be held in the City Art Gallery; It was to Mr. George Murray, and would admit himself "and lady."

"You would?" he said, interpreting aright her rather incoherent remarks, and smiling at her pleasure. "We will go then. You can spare Edith, Frances?"

"I daresay," said Mrs. Lester graciously, "I can do without her for one evening."

Edith ate her breakfast, and rose from the table full of delight. The conversazione was to be in a week, and never was a week so slow in passing.

It did pass, however; the longed for evening came, and Edith stood before her looking glass putting the last touches to her dress. This time she heaved no sigh, felt no dissatisfaction with the sight which met her eyes. Her dress of white muslin and lace was exquisitely fresh and perfectly girlish and simple, but it was made in a way which suited her slender, youthful figure, as nothing else would have done. Her hair, dressed by Mrs. Lester's maid, was coiled loosely yet securely round her head, and blew in little soft curls about her temples; her cheeks were a little flushed, her eyes shone with a soft lustre of happiness, her step was elastic and buoyant; altogether she was as fair an object to look upon as could well be imagined. She smiled to herself as she fastened at her throat some exquisite roses which George had given her to wear. She glanced at the little clock which ticked on the mantelpiece, gave a last look at her own charming reflection, took up her gloves and fan, and went downstairs.

George was already waiting, and the carriage was at the door. Her aunt chid her for being late; but George said, "Nonsense; it did not matter; they had the whole evening before them." And after a glance over her dress—what a different glance from that former one, she thought—he folded her warm wrap round her, and took her out to the carriage.

Long before they reached the Art Gallery, the carriage had to fall into line, and they got on very slowly. Edith did not find fault with the delay. Warmly wrapped in her fur, with her feet on a hot-water tin, she sat by George's side, feeling perfectly happy. She had, to a great extent, lost her shyness with him, and now she talked away merrily.

"You have on my roses, I see," said George, glancing at where they hung at her throat.

"Yes, of course," she answered. "Did you think I should leave them on my dressing-table?"

"You might have forgotten them, or had some from someone else."

"That was exceedingly likely," was her reply, given with a touch of sarcasm which rather alarmed herself, and amused and surprised her companion. "It was so good of you to get them for me," she continued; "they finish off my dress so well, and I had forgotten to get any flowers."

"Ah," he said, with assumed bitterness, "if you only care for them to finish off your dress! I flattered myself you would be pleased because I in particular had given them to you."

"Oh, so I was," she cried, shocked that he should think her ungrateful. "I was very, very glad (she had not lost the youthful habit of repeating her adverbs) you gave them me. I am sure no one else would have thought of such a thing."

"You look very happy," he said after a pause; "I believe you are expecting to enjoy yourself."

"Why, yes; of course I am expecting to enjoy every minute."

"If you determine so much beforehand you are sure to be disappointed; you should always pitch your expectations of pleasure low."

"Is that the right way?" she asked, a little impressed by the seriousness with which he spoke.

"To avoid disappointment, yes; though even that plan is not always successful," he finished, recollecting a recent experience of his own.

After this they were silent, Edith perhaps employed in digesting what he had said; and George's thoughts went back to the subject which had been occupying them much lately. Ever since that evening when he had met Beatrice in little Jack Wilson's home, he had been wanting to atone for his rudeness. That action of hers had influenced his opinion of her greatly, forcing him to reconsider the sweeping judgements which he had hitherto passed on women with the clear conscience of ignorance, and causing him many uncomfortable moments. He hoped to see her to-night. It was chiefly this hope which had brought him, although he was incidentally glad to give pleasure to Edith, and if he did see her, how would she meet him?—with the studied coldness she always showed? How could he overcome this, how wipe away the tacit estrangement between them and win her regard? Filled with these thoughts he forgot the girl by his side, who on her part was occupied solely by thoughts of which he was the beginning and end, and remained plunged in meditations until he was aroused by the arrival of the carriage at the entrance to the Art Gallery.

(*To be continued.*)

### HOMELESS.

With reference to the case of the girl nearly blind, for which a home was wanted, and which was published in The Hospital (p. 95, vol. V.), under this heading, Miss E. Henderson writes: "I beg to thank you for kindly inserting my letter in The Hospital, and to say the result is that through it the poor girl has found what I hope will be a comfortable and permanent home."

